# Rotating Snakes Illusion—Quantitative Analysis Reveals a Region in Luminance Space With Opposite Illusory Rotation

**DOI:** 10.1177/2041669517691779

**Published:** 2017-02-01

**Authors:** Lea Atala-Gérard, Michael Bach

**Affiliations:** Eye Center, Freiburg University, Germany

**Keywords:** motion, illusion, luminance, rotating snakes

## Abstract

The Rotating Snakes Illusion employs patterns with repetitive asymmetric luminance steps forming a “snake wheel.” In the underlying luminance sequence {black, dark grey, white, light grey}, coded as {0, g1, 100, g2}, we varied g1 and g2 and measured illusion strength via nulling: Saccades were performed next to a “snake wheel” that rotated physically; observers adjusted rotation until a stationary percept obtained. Observers performed the perceptual nulling of the seeming rotation reliably. Typical settings for (g1, g2), measured from images by Kitaoka, are around (20%, 60%). Indeed, we found a marked illusion in the region (g1≈{0%–25%}, g2≈{20%–75%}) with a rotation speed of ≈1°/s. Surprisingly, we detected a second “island” around (70%, 95%) with opposite direction of the illusory rotation and weaker illusion. Our quantitative measurements of illusion strength confirmed the optimal luminance choices of the standard snake wheel and, unexpectedly, revealed an opposite rotation illusion.

## Introduction

Certain visual patterns evoke illusory movement, as, for example, in the patterns shown by Fraser and Wilcox (1979). Faubert and Herbert (1999) used very similar patterns and called it the “peripheral drift illusion.” These patterns and their colours were optimised by Kitaoka (2003), resulting in the strong and widely known “Rotating Snakes Illusion.” In 2014, Kitaoka called it “Fraser-Wilcox illusion” (Kitaoka, 2014), but we will use the term “Rotating Snakes Illusion” throughout this article.

The illusion occurs with coloured and grey-shaded patterns (Kitaoka, 2014), and we here consider the grey-shaded version which is easier to parametrise, although the illusion might be stronger when rendered in colour (Backus & Oruç, 2005). Four explanatory models exist (Backus & Oruç, 2005; Conway, Kitaoka, Yazdanbakhsh, Pack, & Livingstone, 2005; Fermüller, Ji, & Kitaoka, 2010; Murakami, Kitaoka, & Ashida, 2006). To further our understanding of the underlying mechanisms, we undertook a quantitative analysis of how the strength of the illusion depends on the luminance levels in the repetitive pattern.

## Methods

The study was approved by our institutional review board (#258/13). Altogether 19 observers (8/11 m/f, 25.7 ± 6.5 years) participated in the experiment with their written informed consent. They were naïve (except the authors) as to the specific experimental question.

**Figure 1.**
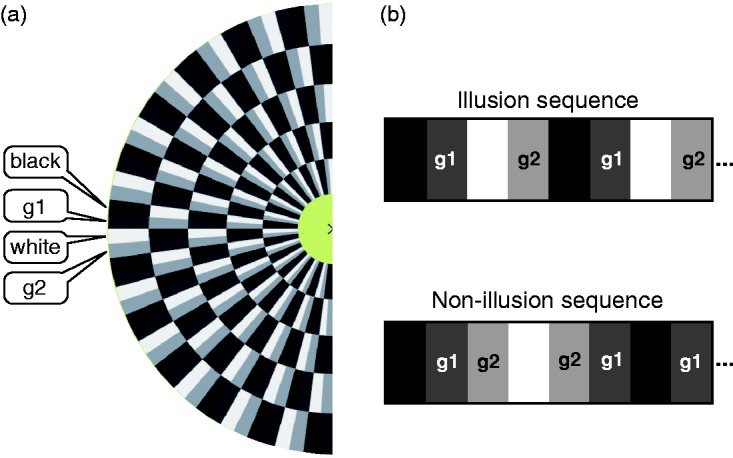
(a) The half greyscale “Snake Illusion disk” shows the two grey fields with luminance levels g1 and g2 adjacent to the black and white anchor fields. (b) Asymmetric sequence that can cause illusory motion (top), and symmetric sequence (the two grey fields are between the black and white fields), thus not evoking illusory motion (bottom).

**Figure 2.**
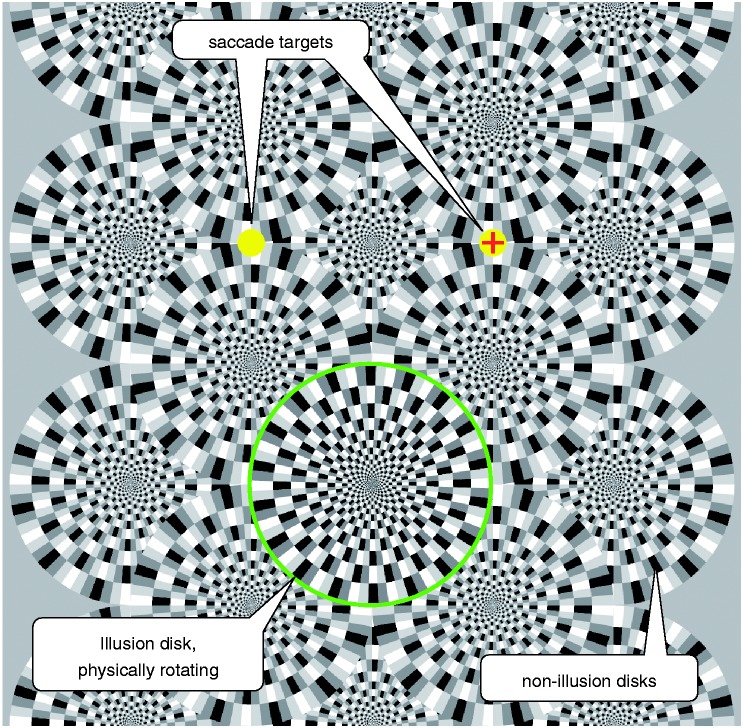
Screenshot of the experimental display. Observers performed saccades (one per second) between the yellow targets. The centre “snake disk” (here encircled in green) rotated physically and was adjusted for perceptual standstill (nulling) by the observer.

The illusory motion depends on asymmetric luminance steps (Kitaoka & Ashida, 2003). Figure 1(a) shows a partial “Snake Illusion disk” and the nomenclature for the four relevant fields: The two black and white anchor fields and the two grey fields with normalised luminances g1 and g2. As a permutation of g1 and g2 results in the same snake disc reflected in space, we present our results only for g1 < g2; the other half of the g1–g2 space can be obtained by mirroring at the diagonal. The full stimulus (Figure 2) consisted of one “illusion disk” (for illustrative purposes here delineated by a green circle), surrounded by many non-illusion disks with the non-illusion pattern-element arrangement as depicted in Figure 1(b) (bottom).

**Figure 3.**
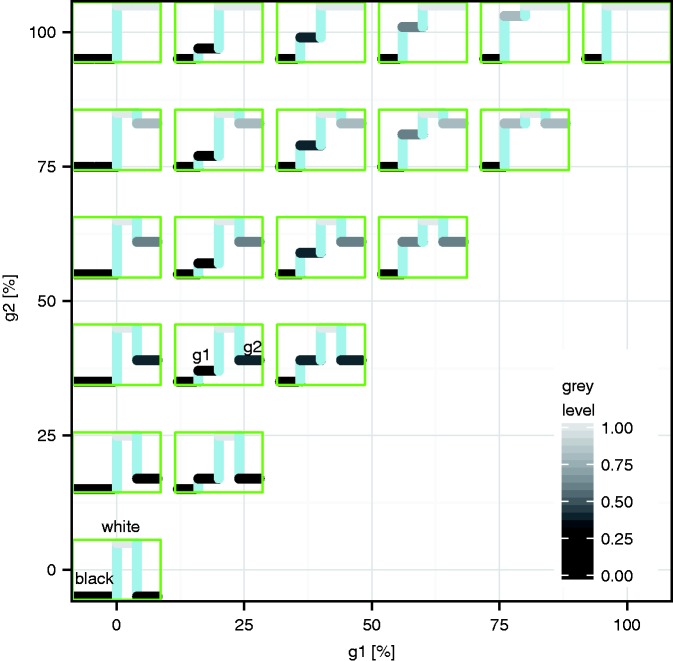
Luminance space of the g1 and g2 levels explored here. Each green subplot depicts one cycle (black-g1–white-g2) of the repeated luminance steps along a “snake disk” (cf. Figure 1).

On a luminance-linearised monitor, the illusion pattern was presented together with two targets (yellow circles) 2.5° left and right of the centre, and 5° above the central illusion disk (the illusion is stronger in peripheral vision). Observers performed saccades between the two yellow targets guided by a red cross with a frequency of 0.5 Hz. For each run, a combination of (g1, g2) values and an initial veridical rotatory velocity was chosen randomly. The observer operated a rotatory button (PowerMate, Griffin, Nashville) until the motion appeared to be nulled (similar to Murakami et al., 2006) and then pressed that button to indicate the end of the trial. Different (g1, g2) values were presented in a random blocked sequence. To prevent a possible build-up of the motion aftereffect, the sequence of (g1, g2) alternated. Figure 3 shows the g1–g2 plane from which we drew (g1, g2) samples. The sign of the rotatory velocity was defined as positive when it was in the direction reported by Backus and Oruç (2005): (black-g1-white-g2). As an intuitive rule, the standard illusory rotation direction is “black towards the darker grey.” In the spirit of reproducible research, original stimulus screenshots, data and analysis scripts (R Development Core Team, 2014) are deposited here: http://dx.doi.org/10.6084/m9.figshare.3804318. When saccading between the yellow targets (Figure 2), a counterclockwise rotation of the encircled disk should be perceived by most readers in this example. Original stimulus screenshots are available here: http://dx.doi.org/10.6084/m9.figshare.3804318.

We here describe three experiments, differing in the number of observers and applied (g1, g2) pairs:

## Results

Our observers found the nulling procedure intuitive and convincing. When they looked directly at the illusion test disk after the trial, they were surprised by its physical rotation.

**Figure 4.**
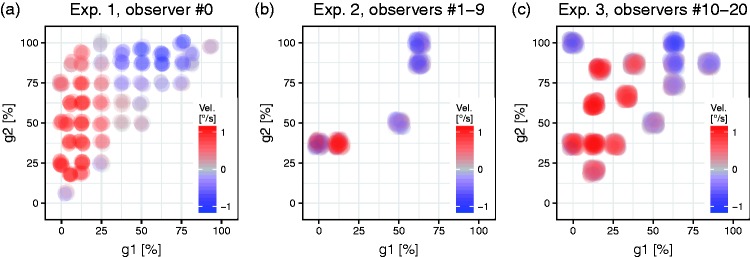
Illusory velocities as function of the luminance levels g1 and g2, arranged like Figure 3. Three experiments differing by number of observers and g1–g2-space sampling. Each run is depicted by a dot, slightly jittered to reveal some of the underlying variability. Red dots (positive velocities) correspond to the “standard” Rotating Snakes Illusion rotation direction, blue dots indicate the opposite direction. Most observers report the “standard” illusion in the left region, and many also perceive an opposite illusion in the “island” at the top around (g1 = 70%, g2 = 95%). See www.michaelbach.de/ot/mot-snakesLum/ for a calibratable demonstration.

In Experiment 1, a single observer #0 (author LAG, Figure 4(a)) sampled the luminance space with high resolution. In the grey-value region (g1 ≈ 20%, g2 ≈ 60%), the expected illusory rotation was clearly observed (reddish). Unexpectedly, however, an opposite illusory rotation obtained in another region of the luminance space, (g1 ≈ 70%, g2 ≈ 95%; blueish). The permutation test resulted in *p* < .001 for g1 and g2.

To verify the unexpected opposite-illusion result, we repeated the experiment with nine additional observers in a down-sampled luminance space (#1–#9, Experiment 2, Figure 4(b)). Most observers indeed needed opposite motion direction for nulling at (g1 ≥ 50%, g2 ≥ 50% > g1). The permutation test found a significance of *p* < .003 for this result.

Experiment 3 aimed to better estimate the shape of the g1–g2-surface of illusion strength (Figure 4(c)). The results are consistent with Experiments 1 and 2: A largish “standard island” peaking at (g1 ≈ 20%, g2 ≈ 60%) and a smaller “opposite island” around (g1 ≈ 70%, g2 ≈ 95%), *p* < .001.

## Discussion

While exploring the effect of luminance combinations in the Snakes Illusion on illusory strength, we discovered a previously undescribed (and weak, but consistent) illusion with opposite rotatory direction for a circumscribed set of luminance combinations. While somewhat interesting in itself, it can serve as a strong test for the four models (Backus & Oruç, 2005; Conway et al., 2005; Fermüller et al., 2010; Murakami et al., 2006). Which of these predict this new finding? A full treatment of this aspect is in preparation, in brief, we have found the following results for this question: The Backus & Oruç (2005) model indeed predicts the opposite illusion for the appropriate (g1, g2) combination but requires a different adaptation curve shape for this. The Conway et al. (2005) model is not compatible with our experimental data. Per this model, no motion is expected in regions where either both g1 and g2 are larger, or both smaller than the average luminance of the pattern (i.e., no reverse-phi motion occurs). Furthermore, in those regions where the above condition does not apply, the model only predicts motion in the classic direction. The Murakami et al. (2006) model does not predict our findings either, since varying the single degree of freedom (which is the imbalance in the temporal derivation operator) could not reproduce our data. We were not able to reproduce the traces published by Fermüller et al. (2010), even when implementing corrected formulae obtained via personal communication, so we need to leave this question open.

## Conclusion

In summary, illusory effect size was largest near the luminance levels (g1, g2) chosen by Kitaoka (2003), namely (20%, 60%) as measured from his image. This maximum region was broad; we found a marked illusion in the region (g1 ≈ {0%–25%}, g2 ≈ {20%–75%}). Surprisingly, however, we detected a second island around (60%, 90%), roughly peaking at (70%, 95%) with an opposite illusory rotation direction (if weak), although the sequence order of g1 and g2 (g1 ≤ g2) stayed the same. Of the four published models for this illusion, the one by Backus and Oruç (2005; assuming a specific adaptation function) is able to predict this finding.

**Table:** 

	Number of participants	Number of (g1, g2) pairs	Number of trials
Exp. 1 (pilot)	1	43	6
Exp. 2	9	5	10
Exp. 3	11	14	10
